# Implicit Learning of Parity and Magnitude Associations with Number Color

**DOI:** 10.5334/joc.428

**Published:** 2025-01-28

**Authors:** Talia L. Retter, Christine Schiltz

**Affiliations:** 1 Institute of Cognitive Science and Assessment, Department of Behavioral and Cognitive Science, University of Luxembourg, Esch-sur-Alzette, LU; 2 Université de Lorraine, CNRS, IMoPA, F-54000 Nancy, FR

**Keywords:** Categorization, Implicit learning, Numerical cognition, Response accuracy, Response time, Visual perception

## Abstract

Associative learning can occur implicitly for stimuli that occur together probabilistically. It is debated whether probabilistic, implicit learning occurs not only at the item level, but also at the category level. Here, we investigated whether associative learning would occur between color and numerical categories, while participants performed a color task. In category-level experiments for each parity and magnitude, high-probability pairings of four numbers with one color were categorically consistent (e.g., the Arabic numerals 2,4,6, and 8 appeared in blue with a high probability, p = .9). Associative learning was measured as higher performance for high-probability vs. low-probability color/number pairings. For both parity and magnitude, performance was significantly better for high- vs. low-probability trials (parity: 3.1% more accurate; magnitude: 1.3% more accurate; 9 ms faster). Category-level learning was also evident in a subsequent color association report task with novel double-digit numbers (parity: 63% accuracy; magnitude: 55%). In control, item-level experiments, in which high-probability pairings were not categorically consistent (e.g., 2,3,6, and 7 appeared in blue with a high probability, p = .9), no significant differences between high- vs. low-probability trials were present. These results are in line with associative learning occurring at the category level, and, further, suggest automatic semantic processing of symbolic numerals in terms of parity and magnitude.

## Introduction

Associative learning is a fundamental channel through which we interpret and interact with our environment, through which stimuli or events that occur together become linked in our minds. Remarkably, associative learning may occur implicitly, as well as with conscious awareness (Reber, 1967; Chun & Jiang, 1998; McClelland, 2006). For example, in an experimental setting, a word appearing consistently in one text color may become implicitly associated with that color, although participants do not report awareness of this; the association may be evidenced through better performance in response to the presented color-word pair than for other pairings (e.g., Pritchatt, 1968; Musen & Squire, 1993). Further, the color-word pair does not need to be presented with a hundred percent consistency to lead to the formation of an association between these items: it is enough that a pair occurs with a high probability. This form of probabilistic associative learning is sometimes referred to as “contingency” or “statistical” learning (Mordkoff, 1996; Saffran, Aslin, & Newport, 1996; Perruchet & Pacton, 2006; Batterink, Paller & Reber, 2019; Schmidt, 2021).

It is possible that implicit, associative learning occurs across stimuli at the category level, as well as at the item level. At the item level, this learning has been well-demonstrated by means of color associations with words, shapes, symbols, etc. (Schmidt et al., 2007; Fecteau, Korjoukov & Roelfsema, 2009; Kusnir & Thut, 2012; Bankieris & Aslin, 2017; Geukes et al., 2019). At the category level, this learning has been suggested through color associations of item categories in only a few studies to our knowledge. Schmidt et al. (2018) reported learning associations of single-exposed exemplars of word categories (animals, professions, and verbs) with their high-probability colors, with a color task. However, effects for high- vs. low-probability trials were small, ranging from 2–11 ms and 0.7–1.8% across two experiments. Recently, Retter, Eraßmy & Schiltz (2023) reported large effects of learning associations of parity categories (even and odd) with high-probability colors (blue and yellow), of 40 ms and 8.3%, with a parity task.

The large effects in the latter study may at least in part relate to participants’ attention being drawn to parity, rather than color, to perform the task. Indeed, category-level learning may be especially prominent when participants attend to generalizable attributes of the stimuli that are salient for task demands (Aslin & Newport, 2012; see also Barsalou, 1990; Newell & Bright, 2002); while color is generalizable, it is readily observed categorically, even without a related task. In contrast, with a color task, the association of words or numbers with the color-correlated category is less obvious, since there are multiple levels or ways in which words or numbers may be categorized. In the case of numbers, they may be categorized in terms of parity, magnitude, primes, multiplication family, etc. However, if people are not asked to attend to, and are not even consciously aware of the relevance of these properties, do they nevertheless perceive numbers in (some of) these terms? This question relates not only to whether implicit, associative learning occurs at a categorical level, but to the automaticity of semantic associations, in particular in the perception of symbolic numbers.

The automaticity of symbolic number representations has been supported by many studies, although there is ongoing debate (e.g., Tzelgov, Porat & Henik, 1997; Guillaume et al., 2020). Evidence in favor of automaticity largely comes from interference paradigms, wherein competition occurs between target and distractor information, e.g., large numbers presented in small fonts or arrays (since Besner & Coltheart, 1979), or across conflicting target-prime categories (e.g., for word magnitude: Dehaene et al., 1998; for numeral parity and magnitude: Reynvoet, Cassens & Brysbaert, 2002). However, the absence of such interference effects when attention is diverted by stimulus factors or task demands is also used to argue against the automaticity of semantic processing of numerals (e.g., Pansky & Algom, 2002; but see Tzelgov et al., 2000). Additionally, it is questioned whether semantic processing of numerals may be limited to the property of magnitude, which may be special in engaging a processing system specific for approximate quantity comparisons (Dehaene, 1992; see also evidence for automatic magnitude processing with spatial associations: Dehaene, Bossini, & Giraux, 1993).

Here, we probe whether color is automatically associated with the concepts of magnitude and parity as derived from symbolic numbers, when participants perform a non-numerical (color) task. We employ an implicit learning paradigm in which high-probability color-number pairings are congruent with parity in one experiment, and with magnitude in another, as compared to non-conceptually grouped numbers (as in Retter, Eraßmy & Schiltz, 2023, but only with a parity experiment and using a parity task). If categorical associations occur between parity and/or magnitude with number color, as predicted, this would be in support of implicit, associative learning occurring at the category level. Additionally, if parity and/or magnitude are associated with color, despite not being explicitly relevant to the participants’ task, this would be in support of the automaticity of semantic associations of parity and magnitude in numerical cognition.

## Methods

### Participants

Participants were human adults recruited from the University of Luxembourg community for a behavioral study in cognitive neuroscience, with no mention of numerical cognition or color. A total of 56 participants were tested, from which two were excluded for low accuracy, below 80%, on control (congruent) trials, leading to a total sample of 54, pseudo-randomly, evenly assigned to the experimental (concept-level) and control (item-level) conditions (age range: 18–30 years; mean age = 22.4 years; 11 male, 1 non-binary; 6 left-handed). All reported normal or corrected-to-normal visual acuity and normal color perception; none reported synesthesia or learning disabilities, including dyscalculia. Their first language of math instruction in school was typically German (French: 6; other: 10). All participants were tested individually, after having provided signed, informed consent for the experiment, which was approved by the Ethical Review Panel of the University of Luxembourg (ERP 21-043), and adhered to the Code of Ethics of the World Medical Association (Declaration of Helsinki, 2013). It may be noted that an original sample size (N = 36 participants) was planned relative to previous experiments reporting substantial implicit associative learning effects (11–16 participants: Fecteau, Korjoukov & Roelfsema, 2009; 13–14 participants per group: Kusnir & Thut, 2012; 19–39 participants: Schmidt & De Houwer 2012; 7 participants without synesthesia: Bankieris & Aslin, 2017; 16 participants per group: Retter, Eraßmy & Schiltz, 2023). However, given feedback from reviews, and in light of small accuracy (1.8%) and response time (11 ms) differences reported in a previous implicit category learning study using a similar color task (Schmidt et al., 2018, Experiment 1; N = 58), we correspondingly increased the sample size (for a consistent pattern of results at N = 36, please see data in Figures S3–S5).

### Stimuli and Materials

The study design followed that of Retter, Eraßmy & Schiltz (2023). Briefly, and including any differences: the stimuli consisted of the Arabic numerals 2, 3, 4, 5, 6, 7, 8, and 9, displayed in Arial font. These numbers were shown at an average height of 1.8° of visual angle, with size variations at each presentation ranging from ±20% of the average size. Font colors were defined by maximal red, blue, green (RGB) representations (out of 255): blue = 0/0/255; yellow = 255/255/0; red = 255/0/0; green = 0/255/0; presented on a background gray = 128/128/128. The experiment was run in PsychoPy3 v2020.2.8 (Peirce et al., 2019), operating on Python (Python Software Foundation, USA). An Acer Spin 3 laptop with a screen size of about 31 × 17 cm and a refresh rate of 60 Hz, was used. Data were processed in Excel (Microsoft, USA) and analyzed with SPSS Statistics 28 (IBM, USA).

### Procedure

An implicit, associative learning paradigm was applied, in which participants were asked to respond to one stimulus dimension (here, stimulus color), and were not informed that this dimension occurs with a high-probability (an average ratio of 10:1) in conjunction with another dimension (number identity). So that associative learning can be assessed, these high-probability trials (termed *congruent* trials) are contrasted with low-probability (*incongruent*) trials in which the number appears in another color.

**Figure 1:**
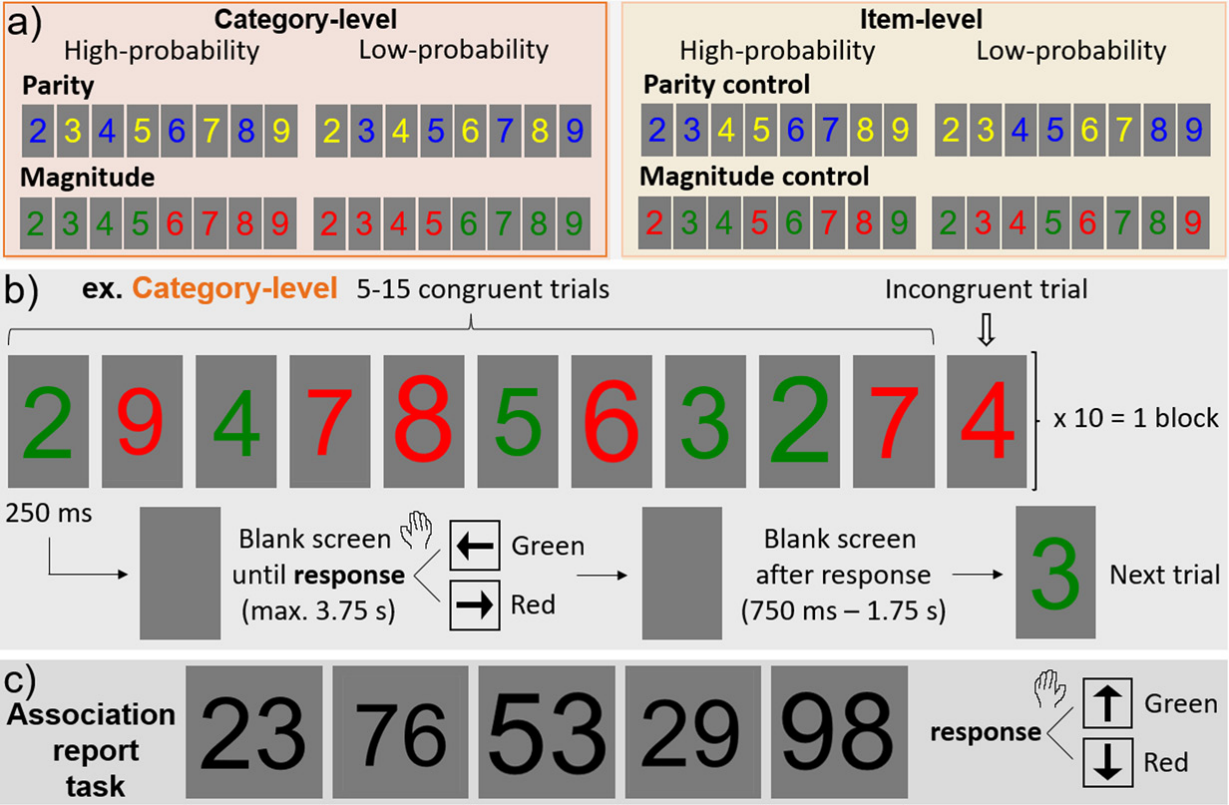
Experimental design. **a)** Parity and magnitude experiments each consisted of two versions: category-level and item-level versions, defined by their high-probability vs. low-probability color-number pairings. **b)** The stimulation paradigm, with an example shown for the category-level version of the magnitude experiment. **c)** Following each main experiment, there was a color association report task with double-digit numbers shown in black font (same trial timing as in panel b; magnitude task shown here).

Two experiments were tested: a parity experiment and a magnitude experiment, each containing two versions (a category-level and item-level version). Each participant took part in both the parity and magnitude experiment, but in only one version (category-level or item-level); in both versions, the order of the parity and magnitude experiment sections was counter-balanced across participants. The parity experiment used only blue and yellow numbers; the magnitude experiment used only green and red numbers. In the parity experiment, the category-level version consisted of high-probability blue-even (2,4,6,8) and yellow-odd (3,5,7,9) number presentations (and thus low-probability yellow-even and blue-odd number presentations; Figure 1a). In the item-level version, the same 8 numbers were assigned with high-probability to the same 2 colors, but without any categorical consistency (high-probability blue: 2,3,6,7; yellow: 4,5,8,9). The magnitude experiment consisted of a category-level version in which small numbers appeared with a high-probability in green (2,3,4,5) and large in red (6,7,8,9); and a categorically inconsistent item-level version (high-probability green: 3,4,6,9; red: 2,5,7,8). Note that magnitude was roughly balanced across high- and low-probability color sets in the parity experiment, and parity was held constant in the magnitude experiment (all sets contained 2 even and 2 odd numbers).

The trial and block design (Figure 1b) were exactly as in Retter, Eraßmy & Schiltz (2023). There were a total of 5 blocks in each the parity and magnitude experiments, each consisting of about 110 trials (10 incongruent and about 100 congruent), i.e., a duration of about 3 minutes. The participants’ task was to respond as accurately and as fast as possible to the number color with the index and middle fingers of their dominant hand, pressing on the arrow keys on the keyboard. The response keys were counter-balanced across participants in each the category-level and item-level experiments: blue/yellow were up/down respectively, or vice versa; and green/red were left/right respectively, or vice versa. This was done to limit the effect of magnitude/spatial biases, e.g., a left arrow response to green, coinciding with a left arrow response to mostly small numbers (see Dehaene, Bossini, & Giraux, 1993).

The main experiment was followed by a parity and a magnitude *association report task* (e.g., Bankieris & Aslin, 2017), in which participants’ task was to explicitly retrieve the color associated with the number stimuli presented in black font (Figure 1c). This experiment was done after both the parity and magnitude experiments, so that participants were not prompted to search for color/number relationships in the second half of the main experiment. Novel, two-digit numbers were used here, to test whether the color associations could extend beyond the exact stimuli previously presented with the same color task. There were 64 trials in each the parity and magnitude association report experiments, consisting of one trial of all the possible combinations of the 8 digits used previously, tested in a single block with a random order. In the parity association report task, participants were given a two-alternative forced choice task to report the color (yellow or blue) that was most associated with the number, with the correct response determined as yellow for odd and blue for even two-digit numbers; in the magnitude association report task, the correct response was determined as green for small (first digit from 2–5) and red for large (first digit from 6–9). Relative to the main experiment, participants responded with their other hand (still using the index and middle fingers) and different response keys (either the up/down or left/right arrows). For example, in the parity experiment participants responded using their dominant hand and the up/down keys, so in the parity association report task they were instructed to respond with their non-dominant hand and the left/right keys. The trial design of the number presentation was otherwise the same as in the main experiment.

Two questions were then asked to the first 36 participants: 1) what did you notice about the experiment; and 2) did you notice that certain numbers usually appeared in a certain color? If so, please give a description. Finally, mathematical fluency was assessed for all participants via the paper-and-pencil *Tempo-Test Rekenen* (TTR; De Vos, 1992). The TTR consists of five sheets of paper, each containing 40 mathematical problems to be solved (1: addition; 2: subtraction; 3: multiplication; 4) division; 5) mixed). Participants were instructed to solve as many problems as possible, in the order given, within a maximum of 1 minute per sheet. The test is scored as the sum of all correctly solved problems, for a maximum of 200. It may be noted that participants’ TTR scores did not differ across the pseudo-randomly assigned groups, *t*_50_ = 0.81, *p* = .43, *d* = 0.22 (concept-level: *M* = 139.5; *SE* = 5.88; item-level: *M* = 133.6, *SE* = 4.41).

### Data analysis

Learning was measured through a potential interference effect of decreased performance for incongruent relative to congruent trials, in terms of increased response time (RT) and/or decreased accuracy. Scores that exceeded 2.5 SDs from the individual’s mean were excluded for correct RTs. Data were analyzed for each accuracy and RT with a repeated-measures analysis-of-variance (ANOVA), with a between-participants factor of experiment *level* (category-level and item-level) and a within-participants factor of *congruency* (congruent and incongruent trials), probing for significant interactions. Planned *t*-tests within each experiment version were also performed to compare congruent and incongruent trials, with one-tailed, paired samples *t*-tests. The color association report task data was analyzed with one-tailed, independent samples *t*-tests. Congruency difference scores, i.e., congruent – incongruent accuracy, and incongruent – congruent RT, were also analyzed with *t*-tests. Correlations were performed after removing any data over 2.5 SDs from the sample means. If Levene’s Test for Equality of Variances was violated, unequal variances’ *t*-tests were applied with corrected degrees of freedom.

## Results

### Category-level

**Figure 2:**
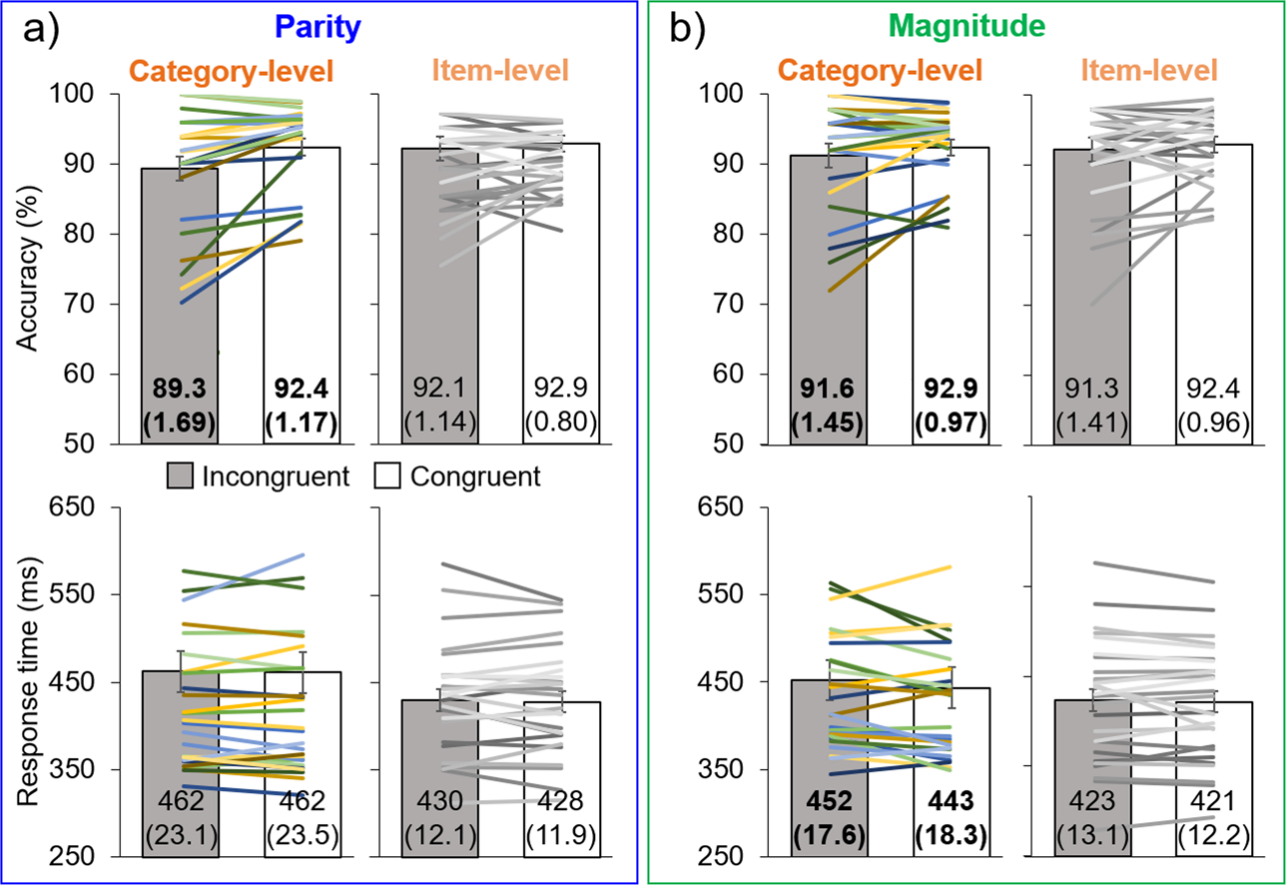
Main experimental results. Results for the parity **(a)** and magnitude **(b)** experiments, in terms of accuracy (top row) and response time (bottom row). Bar graphs represent the means, with error bars of ±1 SE: exact values of means and SE (in parentheses) are given on each bar, with bold font indicating significant differences between incongruent (gray) and congruent (white) trials. The superimposed lines represent individual data, with corresponding color/luminance for individuals’ accuracy and response time.

In the parity experiment, there was lower performance for incongruent (M = 89.3%; SE = 1.69%) than congruent (M = 92.4%; SE = 1.17%) trials in terms of accuracy, i.e., a significant difference of 3.06%, *t*_26_ = 3.67, *p* < .001, *d* = 0.71, as predicted (Figure 2a, upper panel). There was no significant difference in response time (RT) across incongruent and congruent trials (Figure 2a, lower panel), 0.72 ms, *t*_26_ = 0.20, *p* = .42, *d* = 0.037 (incongruent: M = 462 ms, SE = 23.1 ms; congruent: M = 462 ms, SE = 23.5 ms).

In the magnitude experiment, there were small but significant effects in the predicted direction (Figure 2b) for both accuracy, 1.31% (incongruent: M = 91.6%, SE = 1.45%; congruent: M = 92.9%, SE = 0.97%), *t*_26_ = 2.05, *p* = .025, *d* = 0.39, and RT, 8.71 ms (incongruent: M = 452 ms, SE = 17.6 ms; congruent: M = 443 ms, SE = 18.3 ms), *t*_26_ = 1.76, *p* = .045, *d* = 0.34.

### Item-level

In the parity experiment, there were no significant differences in terms of accuracy between incongruent (M = 92.1%; SE = 1.14%) and congruent trials (92.9%; SE = 0.80%), 0.73%, *t*_26_ = 0.94, *p* = .18, *d* = 0.18, or in terms of RT (incongruent: M = 430 ms; SE = 12.1 ms; congruent: M = 428 ms; SE = 11.9 ms), 1.90 ms, *t*_26_ = 0.53, *p* = .30, *d* = 0.10.

In the magnitude experiment, there were also no significant differences in terms of accuracy between incongruent (M = 91.3%; SE = 1.41%) and congruent trials (M = 92.4%; SE = 0.96%), 1.12%, *t*_26_ = 1.17, *p* = .13, *d* = 0.22, or in terms of RT (incongruent: M = 423 ms; SE = 13.1 ms; congruent: M = 421 ms; SE = 12.2 ms), 1.91 ms, *t*_26_ = 0.63, *p* = .27, *d* = 0.12.

### Cross-level interactions

There was only a significant interaction between experiment *level* and *congruency* for the parity experiment in terms of accuracy, *F*_1,52_ = 4.17, *p* = .046, ɳ_p_^2^ = 0.074, demonstrating a strong congruency effect for the category-level experiment only (3.06% effect at the category-level *vs*. 0.73% at the item-level). There was a significant main effect of congruency, *F*_1,52_ = 11.09, *p* = .002, ɳ_p_^2^ = 0.18, and no main effect of *level, F*_1,52_ = 0.995, *p* = .32, ɳ_p_^2^ = 0.019. There were no significant main effects or an interaction in terms of RT, all F’s < 1.7, p’s > .2, ɳ_p_^2^’s < 0.031.

For accuracy in the magnitude experiment, there was a significant main effect of *congruency, F*_1,52_ = 4.68, *p* = .035, ɳ_p_^2^ = 0.083, and there was no main effect of *level, F*_1,52_ = 0.082, *p* = .78, ɳ_p_^2^ = 0.002, or an interaction, *F*_1,52_ = 0.093, *p* = .76, ɳ_p_^2^ = 0.002. The statistical results were similar in terms of RT: *congruency, F*_1,52_ = 3.36, *p* = .072, ɳ_p_^2^ = 0.061; *level, F*_1,52_ = 1.36, *p* = .25, ɳ_p_^2^ = 0.025; interaction, *F*_1,52_ = 1.38, *p* = .25, ɳ_p_^2^ = 0.026.

### Experiment blocks

**Figure 3:**
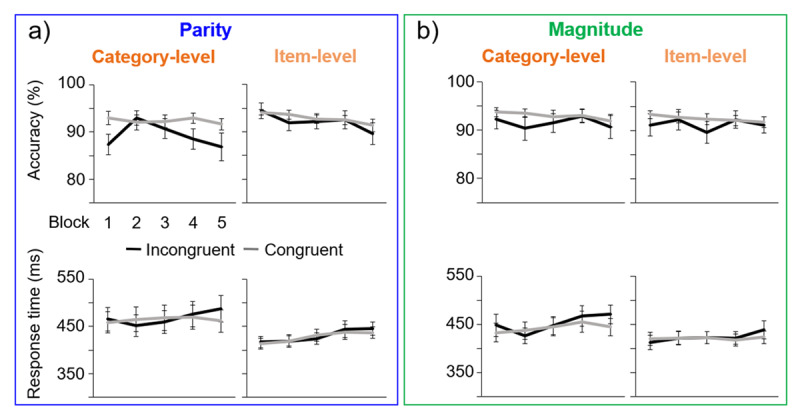
Mean results across blocks (1–5), with error bars of ±1 SE. **a)** The parity experiment, for both category-level and item-level experiment levels. **b)** The magnitude experiment.

The evolution of responses to congruent and incongruent trials was examined across the five experimental blocks (Figure 3). There appeared to be general trends for decreasing accuracy and increasing response time for incongruent trials across blocks in both parity and magnitude experiments at the category-level, examined in detail below.

#### Parity

For parity accuracy in the category-level experiment, the main effect of *congruency* was present, *F*_1,26_ = 10.1, *p* = .004, ɳ_p_^2^ = 0.28, as incongruent accuracy was lower overall. There was not a main effect of *block, F*_4,23_ = 1.96, *p* = .14, ɳ_p_^2^ = 0.25, but the interaction between *congruency* and *block* bordered on significance, *F*_4,23_ = 2.54, *p* = .067, ɳ_p_^2^ = 0.31, as only the incongruent accuracy tended to decrease across blocks. Paired-samples t-tests showed significant differences between congruent and incongruent trials in the first, fourth, and nearly fifth blocks (all *t*’s > 1.6, *p*’s < .053, *d*’s > 0.31), but not in the second or third block (*t*’s < 1.2, *p*’s > .12, *d*’s < 0.23).

Regarding RT, there was a significant interaction between *congruency* and *block*: *F*_4,104_ = 3.38, *p* = .012, ɳ_p_^2^ = 0.12 (no main effects: *congruency*: *F*_1,26_ = 1.04, *p* = .32, ɳ_p_^2^ = 0.038; *block*: *F*_2.5,65.3_ = 0.78, *p* = .49, ɳ_p_^2^ = 0.029), as the RT increased across blocks only for incongruent trials. A significant difference emerged between congruent and incongruent trials only in the fifth block, t_26_ = 2.54, p = .009, d = 0.49), (all other *t*’s < 1.6, *p*’s > .07, *d*’s < 0.3).

#### Magnitude

For magnitude accuracy in the category-level experiment, there were no significant main effects, nor an interaction (*congruency* nearing significance: *F*_1,26_ = 3.80, *p* = .062, ɳ_p_^2^ = 0.13; *block*: *F*_4,72.4_ = 0.52, *p* = .66, ɳ_p_^2^ = 0.020; interaction: *F*_4,104_ = 0.46, *p* = .76, ɳ_p_^2^ = 0.017).

In terms of RT, there were also not significant main effects (*congruency*: *F*_1,26_ = 2.69, *p* = .11, ɳ_p_^2^ = 0.094; *block*: *F*_2.4,62.5_ = 2.37, *p* = .092, ɳ_p_^2^ = 0.084), but there was a significant interaction, *F*_3.77,98.1_ = 3.27, *p* = .016, ɳ_p_^2^ = 0.11), as RT increased in the final blocks only for incongruent trials. Paired-samples t-tests revealed significant differences between congruent and incongruent trials in the first and fifth blocks (*t*’s > 2.0, *p*’s < .029, *d*’s > 0.38), but not in the other blocks (*t*’s < 1.36, *p*’s > .093, *d*’s < 0.27).

### Association report task

#### Parity

**Figure 4:**
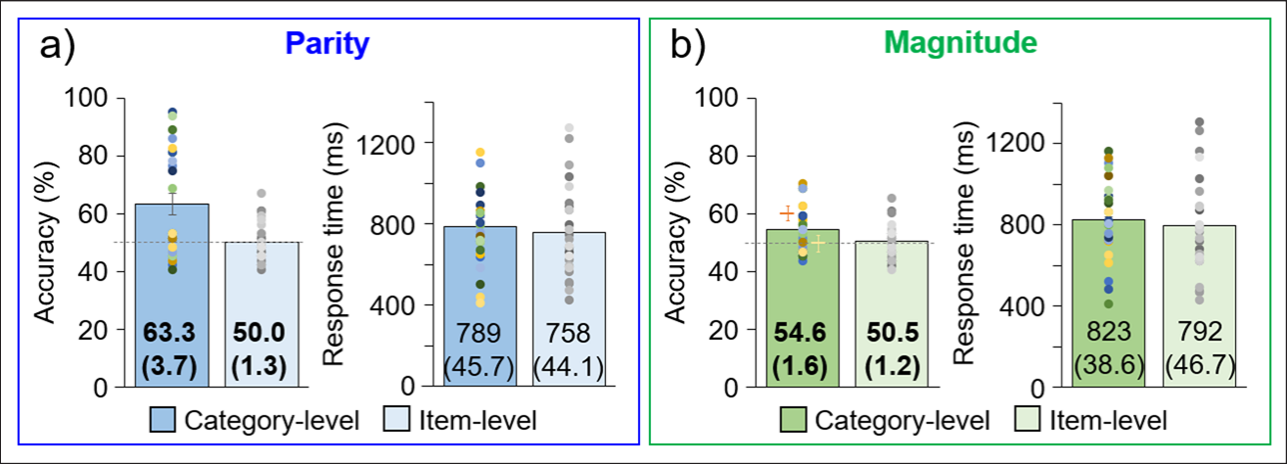
Explicit, color association report task results. Individual participants are represented as dots above the group-level mean bar plots, with error bars of ±1 SE (exact M and (SE) values are given on the bars). Bold font indicates a significant difference between category- and item-level experiments. Chance level accuracy is indicated with a horizontal gray line. **a)** Parity experiment. **b)** Magnitude experiment. In the magnitude experiment, the orange and yellow plus signs indicate the accuracy for congruent and incongruent double-digit numbers, respectively.

For the category-level experiment version, participants reported their color-association consistent with the color-parity pairing in the main experiment with 63.3% accuracy (SE = 3.69%), while for the item-level experiment this was at chance, 50.0% (SE = 1.34%) (Figure 4a). The difference in accuracy across experiment levels was significant, *t*_30.2_ = 3.37, *p* = .001, *d* = 0.94. Regarding RT, there was not a significant difference across experiment levels, *t*_49_ = 0.49, *p* = .31, *d* = 0.14 (category-level M = 789 ms; SE = 45.7 ms; item-level M = 758 ms; SE = 44.1 ms).

#### Magnitude

The color-association was reported as 54.6% (SE = 1.64%) consistent with the color-magnitude pairing in the main experiment at the category-level, but only 50.5% (SE = 1.18%) for the item-level, a significant difference, *t*_45.3_ = 2.03, *p* = .024, *d* = 0.56 (Figure 4b). At the category-level, it was observed that the accuracy was higher for congruent double-digit numbers, in which the second digit was congruently small or large with the first digit, than for incongruent numbers (congruent: M = 60.4%; SE = 2.31%; incongruent: M = 49.9%; SE = 2.72%); no differences for the item-level experiment (congruent: M = 51.0%; SE = 2.20%; incongruent: M = 51.0%; SE = 1.41%). As for the parity experiment, the response time was not significantly different across experiment levels, *t*_50_ = 0.52, *p* = .30, *d* = 0.14 (category-level M = 823 ms; SE = 38.6 ms; item-level M = 792 ms; SE = 46.7 ms).

### Correlations

Correlations were explored between accuracy in the explicit, color association report task and significant congruency effects in the main experiment. In the parity experiment, individuals’ association report accuracy was positively, significantly correlated with their congruency difference accuracy scores (congruent – incongruent), *r*_24_ = .43, *p* = .037, in the category-level experiment (Figure S1). In the magnitude experiment, individuals’ association report accuracy did not significantly correlate with their congruency difference accuracy scores, *r*_24_ = .24, *p* = .25, or RT scores (incongruent – congruent), *r*_15_ = .005, *p* = .98, in the category-level experiment.

### Questionnaire

In response to being asked what they noticed about the experiment, some participants at both experiment levels reported seeing colors and numbers, just mentioning colors, or just mentioning numbers. They also sometimes reported seeing the variations in stimulus presentation, in terms of timing and size. When asked if they noticed any numbers usually appearing in a certain color, in the category-level experiment, four participants reported noticing 2–4 specific color/number relationships as follows, per participant: 7 mostly in green and 3 mostly in red (both incorrect); 3 and 5 in yellow; 2 mostly in green and 6 mostly in blue or red; 5 in green, 7 in red and blue (incorrect), and 3 in yellow. In the item-level experiment, eight participants reported noticing 1–6 specific color/number relationships. On average, the reported pairings in the category-level experiment were 73% accurate, as compared to 58% in the item-level experiment.

Importantly, only two participants reported anything about parity in the category-level experiment: one participant reported that odd numbers appeared in yellow and even in blue; the other reported that at the end of the experiment odd numbers mostly appeared in yellow and even mostly in blue. Surprisingly, two participants also reported a parity/color relationship in the item-level experiment: one for blue with odd, and yellow with even; the other for even with blue and green, and odd with red and yellow. Five participants reported something about magnitude in the category-level experiment, including the two that had observed parity, although no description was completely accurate. (It is worth noting that removing the participants who reported noticing parity (N = 2) and magnitude (N = 5) in the category-level experiment from the analysis would not have changed the results (Figure S2). For parity, this would have been a 0.0% change in the accuracy effect and a 2 ms decrease in the RT effect. For magnitude, this would have been a 1.0% decrease in the accuracy effect but a 6 ms increase in the RT effect. Further, the association report accuracy would only have been reduced by a few percentage points in each experiment (parity: 2.2%; magnitude: 2.6%).

One participant reported that higher numbers were red and yellow and lower numbers were blue and green; another reported 2–6 more green and 7–9 more red. Three participants reported effects split around the number 5; specifically, these participants described: numbers above 5 in red and below 5 in green; numbers above 5 and below 5 each having a certain color most of the time, but giving an example of changing color between blue and yellow; and that at the end of the experiment 6–9 mostly appeared in red and 1–4 mostly in green. No participants in the item-level experiment reported a magnitude/color relationship.

## Discussion

### Category-level learning

The results are in line with implicit, associative learning occurring at the category level. This was primarily evidenced by higher performance for congruent than incongruent trials within the category-level experiment: for parity, accuracy was on average 3.1% higher (and RT insignificantly 1 ms faster) for congruent *vs*. incongruent trials; for magnitude, accuracy was 1.3% higher and RT 9 ms faster. These effects emerged over the course of the experiment, as participants formed associations of numerical concepts and colors. Further in line with a category advantage, in the association report task, item-level participants were at about 50% accuracy, i.e., not above chance, but above-chance accuracy was reported for the category-level experiment (parity: 63%; magnitude: 55%).

For parity, the accuracy effect cannot be explained by item-level associations of individual numbers and colors: in the item-level experiment version, there were substantially smaller, insignificant differences in accuracy, of 0.7%, leading to a significant interaction of experiment level and congruency. Although only present in the accuracy measure, this provides strong support of an advantage of implicit, associative learning of parity-color associations at the category level over the item level. In regards to magnitude, there were no significant interactions across experiment level and congruency, limiting the support of a category-level advantage. However, there was significantly higher performance for congruent than incongruent trials within the category-level experiment only: again, accuracy was 1.3% higher and RT 9 ms faster at the category level, compared to 1.1% and 2 ms at the item level. This represents a very small advantage in accuracy at the category level, but over a four times larger effect in terms of RT. This evidence of category-level learning complements reports from previous studies using probability-based paradigms (Schmidt et al., 2018; Retter, Eraßmy & Schiltz, 2023; see also Schmidt & De Houwer, 2012, as well as from other associative/priming studies using categorical stimuli: Schacter & Graf, 1986; Hartman, Knopman & Nissen, 1989; Lambert & Sumich, 1996; Goschke & Bolt, 2007; Brady & Oliva, 2008; Schmidt et al., 2018).

It should be noted that the level of difficulty for learning color/number associations was greater for the item-level than category-level experiment: there were eight pairings of individual numbers and colors in each item-level experiment, but this could be reduced to two (blue/even and yellow/odd; or green/small and red/large) in each category-level experiment, if category-level associations were formed. This could be taken as there being about 250 congruent category/color presentations across each experiment, but only about 63 congruent number/color presentations (with congruent learning being countered by additional, occasional incongruent presentations in both cases). Thus, the lack of significant item-level learning here, despite minor trends in the predicted direction across congruent and incongruent trials, can likely be attributed to inadequate opportunity to learn a greater number of associations in the item-level experiment version, rather than suggesting that item-level learning does not occur (e.g., item-level associative learning of 3–4 pairings with sessions spanning 2–4 days: Kusnir & Thut, 2012; Bankieris & Aslin, 2017; Geukes et al., 2019; but see also Schmidt et al., 2007 with 72 congruent presentations of 4 learned pairings). However, a reduction of complexity due to category-level learning is likely not an experimental artifact, but may facilitate (long-term) associative learning in the natural environment (e.g., that male voices are associated with male rather than female faces: Barenholtz et al., 2014). Overall, category-level learning may be interpreted as occurring in addition to item-level associative learning, which may occur at the level of sensation/perception and/or at the response level (e.g., Colzato, Raffone, & Hommel, 2006; Conway & Christiansen, 2006; Schmidt et al., 2007).

The effects reported here, with a color task, are much lower than in a previous, parallel experiments reporting implicit associations of color with a parity task: 8.3% and 40 ms at the category-level (Retter, Eraßmy & Schiltz, 2023). This is not surprising, however, because the large effects in the latter study may at least in part relate to participants’ attention being drawn to the more abstract category, parity, to perform the task, while the categorical color information of the stimuli was still readily observed. On the other hand, with a color task here, participants’ attention is drawn to a readily observable stimulus property, and not on (one of many possible) conceptual numerical categories. Again, attention to generalizable attributes, salient for task demands, has previously been demonstrated to strengthen category-level learning (Aslin & Newport, 2012; see also Barsalou, 1990; Newell & Bright, 2002). The small effects reported here could nevertheless benefit from replication in future studies.

The mechanism for probabilistic, implicit, associative learning may depend on linking the neural representation of one stimulus attribute with the other paired stimulus attribute, in line with Hebbian learning (Hebb, 1949; McClelland, 2006). Indeed, it may be a general principle that the perception of a stimulus evokes the representation of its associated attributes; and this may be the case not only for perceptual and/or motor associations, but for conceptual associations as well (e.g., Martin, 2007; Purves, 2019). For specific examples: the Stroop effect has been demonstrated for color-associated words (e.g., sky or lemon; Klein, 1964; Dalrymple-Alford, 1972; Risko, Schdmit & Besner, 2006); and synesthetic color associations may be evidenced across different numerical representations/equations (Dixon et al., 2000; Ramachandran & Hubbard, 2001; Ward et al., 2007). Following this premise, category-level learning may not be limited to implicit, associative learning, but rather be a general principle of cognitive functioning more generally (Jacoby & Brooks, 1984; Edelman, 1992; Knowlton, & Squire, 1993; Barsalou, 2008).

### Implicit learning questionnaire

The associative learning between numerical categories and color arose with most participants being unaware of the category/color relationships occurring with a high probability. From the 36 participants completing the questionnaire, only two reported noticing a color correspondence with parity, and five participants with magnitude, in the category-level experiment, although these descriptions were not fully accurate. However, removing these participants from the analysis did not appreciably change the results (Figure S2). Thus, despite some participants reporting explicit awareness of the category/color pairings, overall comparable associative learning as produced with participants unaware of these manipulations (e.g., as also in Jiménez, Mendez & Cleermans, 1996; Chun & Nakayama, 2010; Schmidt et al., 2007; Schmidt & De Houwer, 2012; Geukes et al., 2019; for a review: Schmidt, 2021).

A lack of explicit awareness driving the congruency effects implies that implicit learning is sufficient to explain the results. The presence of above-chance accuracy in the association report task for both parity and magnitude at the category-level does not contradict this: participants can do this task without being aware of a reason for why they choose one color over another (see Jiménez, Mendez & Cleermans, 1996). Indeed, if participants followed explicit parity or magnitude rules for this association report task, their performance would be expected near ceiling, while in practice the highest accuracy reported for any individual participant completing the questionnaire was below 90% (parity: 89%; magnitude: 70%; and in both cases, this participant being unaware of a category/color relationship).^1^

This learning cannot be explained by response learning (of a number or color with a response key/motor activity), because participants used a different hand and response keys for their responses than in the main experiment (see Geukes et al., 2019). Moreover, it did not arise due to perceptual stimulus characteristics since the stimuli used in this association task were novel, double-digit stimuli, requiring an abstraction of conceptual information.

### Automaticity of semantic associations for symbolic numbers

If parity and magnitude were implicitly associated with color, without participants awareness of the high-probability color associations, and even without directed attention to a numerical property, this would be in line with an automatic processing of semantic associations for symbolic numerals. Such a finding would be in line with many previous studies, with a range of approaches, particularly for magnitude processing (e.g., Besner & Coltheart, 1979; Dehaene, Bossini, & Giraux, 1993; Reynvoet, Cassens & Brysbaert, 2002). It is intuitive to suppose that an automatic processing of magnitude symbols may be mapped onto to a (possibly innate) system of quantity relations (see Dehaene, 1992). At a neural level, this may be supported by topographically ordered responses to quantities in the human cortex (Harvey et al., 2013). Automatic processing of magnitude may thus arise through associations of symbolic numbers and relative quantities, with distinct neural representations for “small” and “large” quantities, despite these categories not being precisely defined (indeed, most participants aware of a magnitude/color relationship here reported a category division around 5, although numbers were actually divided between 5 and 6; moreover, their above-chance accuracy for double-digit numbers was based on relative magnitude as determined by the first digit). However, the lack of a significant interaction across concept-level and item-level experiments here limited the support of automatic magnitude processing, despite significant associative leaning effects in both accuracy and RT, and above-chance association-report accuracy, only in the category-level experiment. One reason for limited support of automatic magnitude processing here may indeed be that large and small numbers are artificially defined as categories here, rather than being allowed to exist on a continuum (e.g., see Leibovich et al., 2017).

The automaticity of parity processing has remained more controversial (Dehaene, 1992; Guillaume et al., 2020; for evidence in favor: Shepard et al., 1975; Miller & Gelman, 1983; Reynvoet, Cassens & Brysbaert, 2002; Fabre & Lemaire, 2005; Retter, Eraßmy & Schiltz, 2024; see also Krueger & Hallford, 1984). However, parity/color associations here were not weaker than magnitude/color associations; indeed, only for the parity experiment did a significant interaction occur between experiment level and congruency. Theoretically, parity is essentially a binary category that may be well-suited to assignment to two distinctive color categories (although even may represent a more salient category than odd, and some numbers may be perceived as more even/odd than others: e.g., Hines, 1990; Heubner et al., 2018). It is also possible that even for parity, a more abstract numerical property, automatic processing is also supported by associations of parity with distinctive neural representations, such as for symmetry/asymmetry (Bertamini et al., 2018).

### Color as a task-relevant, associated dimension

The color discrimination task here was very easy, and there is no reason to think that participants would rather focus on parity or magnitude to perform it: indeed, participants who were explicitly aware of the color/category manipulations did not have greater effects than those who were unaware (Figure S2). While having a task that relates to the probabilistic congruency of parity and magnitude might be considered as a weakness for implicit learning (Besner, Stolz & Boutlier, 1997; but also Shanks & St. John, 1994), it could also be argued that a non-numerical task serves as a distractor for participants, as opposed to passive viewing, limiting their resources for looking for an explicit contingency in the numerical stimuli (Svartdal, 1994). Anecdotally, two participants in the category-level, and three participants in the item-level experiment spontaneously reported looking for a pattern during testing. Whether or not the color task was necessary for associative learning here cannot be directly addressed, although previous studies with secondary or dual tasks have still reported implicit learning effects (see Stadler, 1995). In numerical cognition research, a color task has also successfully been used in order to measure the spatial-numerical association of response codes, with a non-numerical task (Keus & Schwarz, 2005; Bull, Cleland & Mitchell, 2013; Hoffmann et al., 2013; but no effect in Fias, Lauwereyns & Lammertyn, 2001).

Here, distinctive color pairs were assigned to contrast numerical categories: blue and yellow with even and odd and red and green with large and small (Webster, 2020), which may bolster the association of color with conceptual categories (e.g., there was a categorical effect in the word-reading Stroop task in Smithson et al., 2006). The colors used here were also represented with reasonably prototypical exemplars, at high intensity, and not controlled for luminance or saturation, which may have contributed to the saliency of the “color” associations (see Jameson & D’Andrade, 1997; and for a prototypical bias of memory colors: Bartleson, 1960; Bae et al., 2015). Previous studies investigating implicit learning have also used reasonably prototypical color exemplars, although usually with a larger number of less distinctive colors (e.g., blue, yellow, green and orange: Schmidt et al., 2007; blue, yellow, green, red, cyan, and magenta: Kusnir & Thut, 2012). It is possible that color and numbers, or color in particular, are especially well-suited for establishing associations (see the discussion of Retter, Eraßmy & Schiltz, 2023).

## Data Accessibility Statement

Data (anonymized behavioral data for the 54 human participants included in the sample) are publicly available on the Zenodo repository: https://doi.org/10.5281/zenodo.14710403.

## Additional File

The additional file for this article can be found as follows:

10.5334/joc.428.s1 Supplemental Figures. Figures S1 to S5.

## Footnotes

Although, participants were not entirely correct in their explicit realizations: especially for magnitude, no participant correctly observed that numbers 2–5 were most often green and 6–9 most often red, instead most reported a split around 5. Similarly, reports were often inaccurate (73% accuracy) for the individual number/color relationships in the category-level experiment.
