# Supplementary material for: Implicit Learning of Parity and Magnitude Associations with Number Color
Source: J Cogn. 2025 Jan 28;8(1):21. doi: 10.5334/joc.428 (PMC11784500; doi:10.5334/joc.428)
Supplement: Supplemental Figures. — Figures S1 to S5. [file joc-8-1-428-s1.pdf]

Supplemental figures

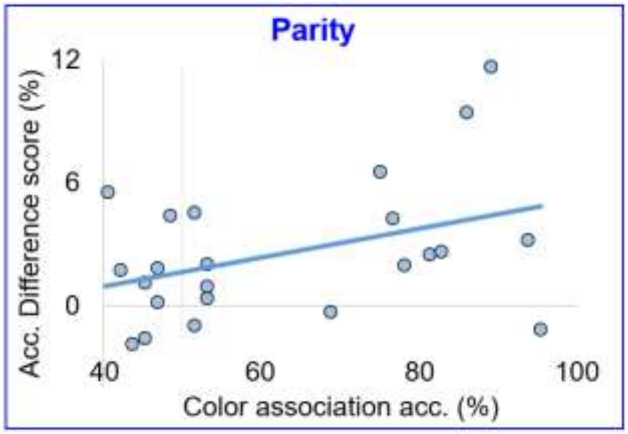

Figure S1. *Color association report accuracy correlated with the accuracy effect in the main experiment for concept-level parity.*

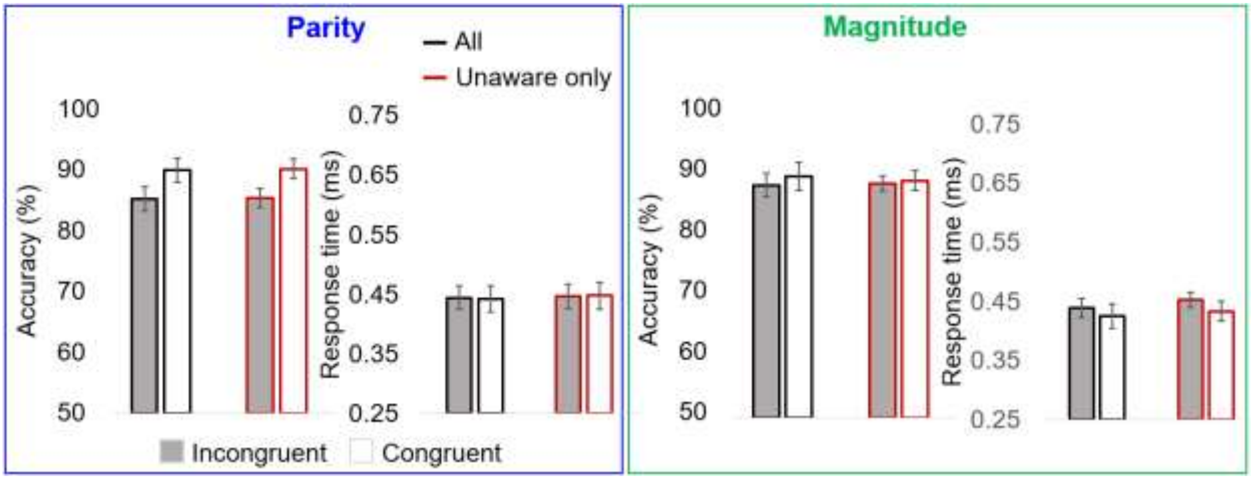

Figure S2. Questionnaire results impact (N=36). *Main concept-level experimental results for participants unaware of the congruency between color and numerical concepts (bars outlined in red; parity: N=15; magnitude: N=12), contrasted to all the participants (bars outlined in black; parity and magnitude: N=17). Bar graphs display the means, with error bars of ±1 SE.*

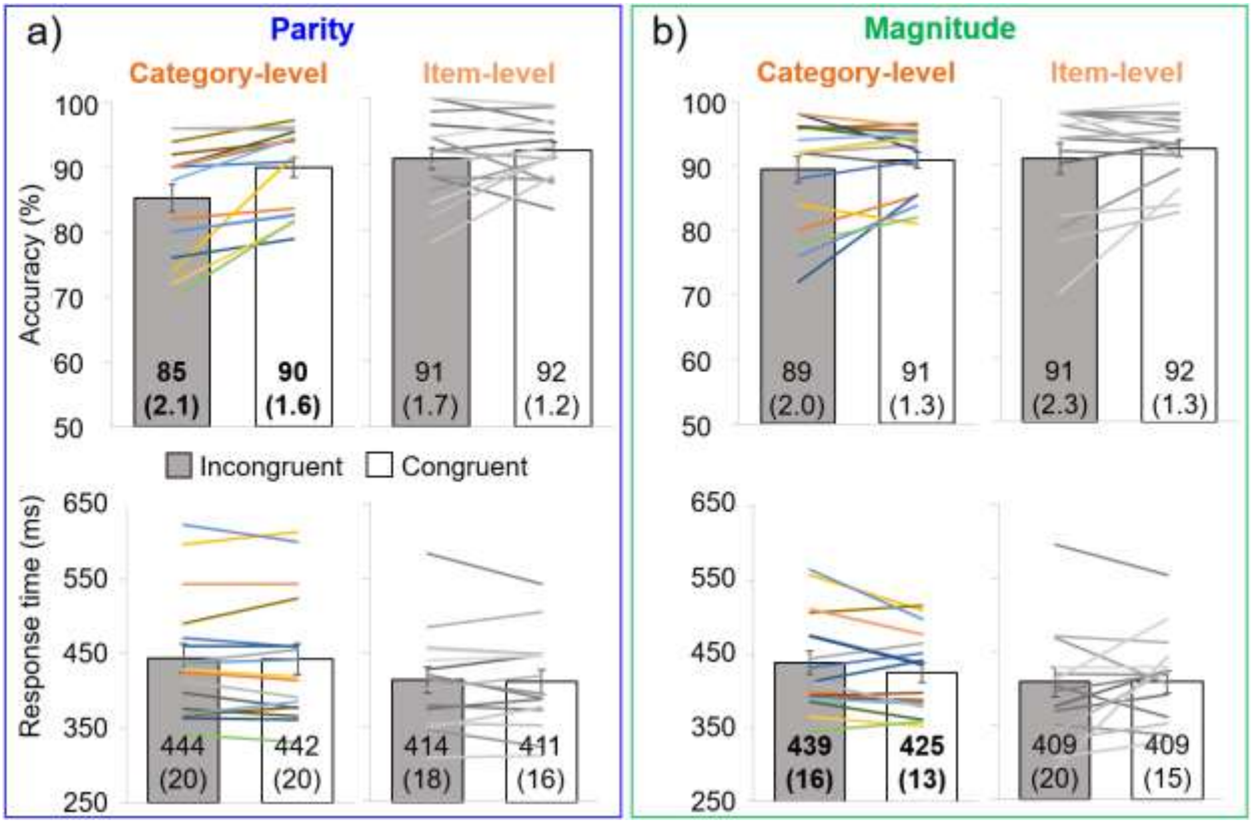

Figure S3. *Main experimental data for the first 36 participants (notes as in Fig. 2).*

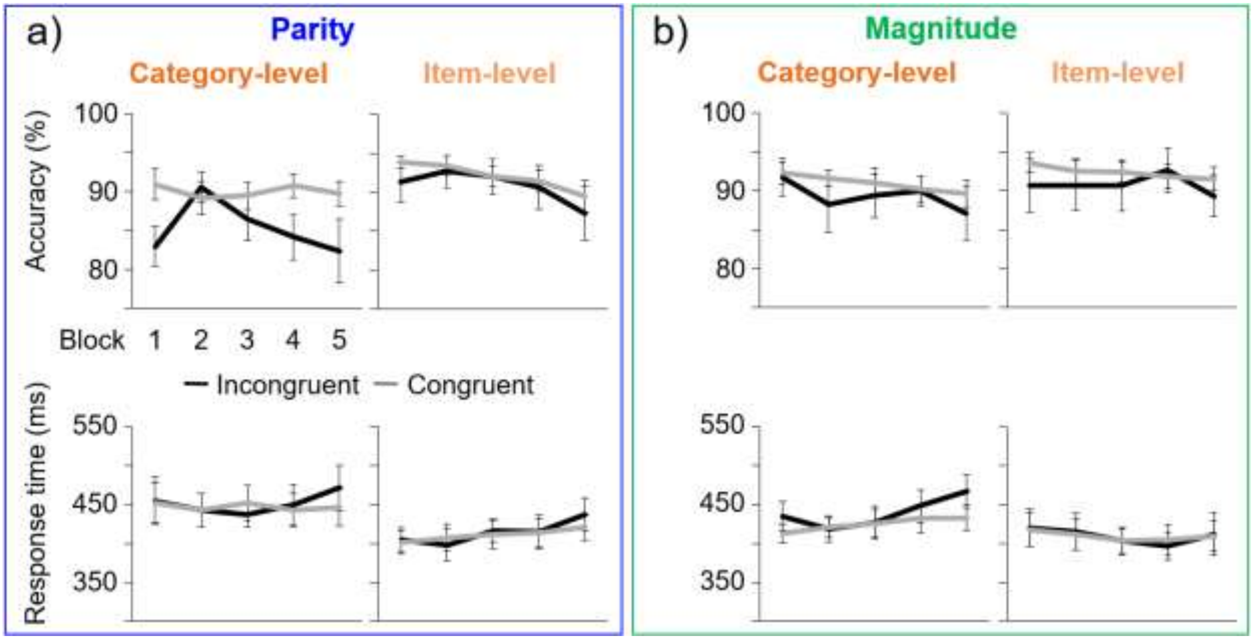

Figure S4. *Mean results across blocks (1-5) for the first 36 participants (notes as in Fig. 3).*

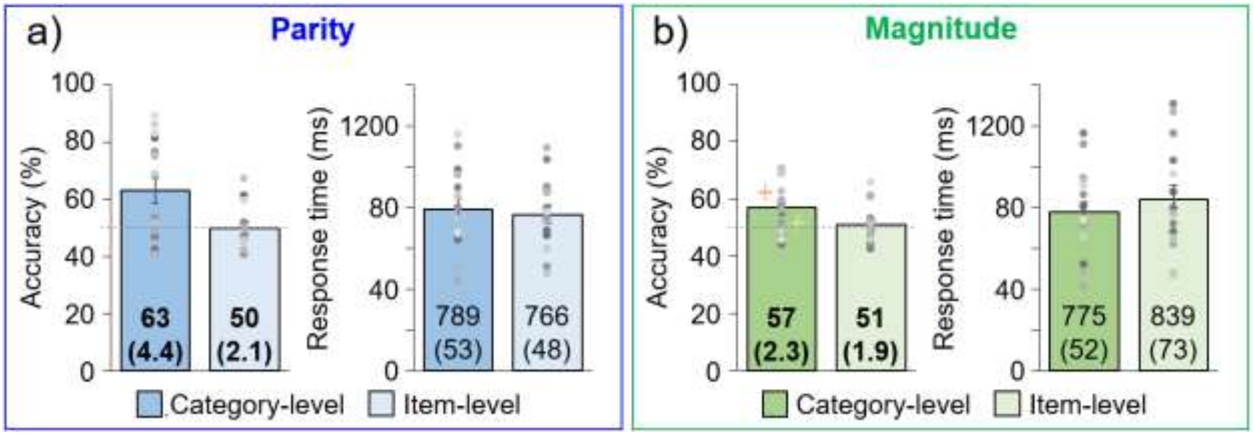

Figure S5. *Explicit, color association report task results for the first 36 participants (notes as in Fig. 4).*
